# Hypothetical COVID-19 protection mechanism: hints from centenarians

**DOI:** 10.1186/s12979-021-00226-z

**Published:** 2021-03-30

**Authors:** Franca Rosa Guerini, Matteo Cesari, Beatrice Arosio

**Affiliations:** 1 IRCCS Fondazione Don Carlo Gnocchi, ONLUS, Milan, Italy; 2 Geriatric Unit, IRCCS Istituti Clinici Scientifici Maugeri, Milan, Italy; 3 Department of Clinical Sciences and Community Health, University of Milan, Milan, Italy; 4 Geriatric Unit, Fondazione IRCCS Ca’ Granda Ospedale Maggiore Policlinico, Milan, Italy

**Keywords:** Aging, COVID-19, Frailty, Centenarians, HLA

## Abstract

The risk of serious complications and the fatality rate due to COVID-19 pandemic have proven particularly higher in older persons, putting a further strain in healthcare system as we dramatically observed.

COVID-19 is not exclusively gerophile (géro “old” and philia “love”) as young people can be infected, even if older people experience more severe symptoms and mortality due to their greater frailty. Indeed, frailty could complicate the course of COVID-19, much more than the number of years lived. As demonstration, there are centenarians showing remarkable capacity to recover after coronavirus infection.

We hypothesize that centenarian’s portfolio could help in identifying protective biological mechanisms underlying the coronavirus infection.

The human leukocyte antigen (HLA) is one of the major genetic regions associated with human longevity, due to its central role in the development of adaptive immune response and modulation of the individual’s response to life threatening diseases. The HLA locus seems to be crucial in influencing susceptibility and severity of COVID-19.

In this hypothesis, we assume that the biological process in which HLA are involved may explain some aspects of coronavirus infection in centenarians, although we cannot rule out other biological mechanisms that these extraordinary persons are able to adopt to cope with the infection.

## Presentation of the hypothesis

The risk of serious complications and the fatality rate due to COVID-19 pandemic have proven particularly higher in older persons, putting a further strain on healthcare system.

Globally, at 3 March 2021, there have been 114,315,846 confirmed cases of COVID-19, including 2,539,427 deaths, reported to World Health Organization (WHO Coronavirus Disease (COVID-19) Dashboard).

In Italy, the most long-lived country in Europe, the mean age of patients dying for SARS-CoV-2 infection was 81 years (median 83, range 0–109, IQR 75–88). Women were 37,295 (43.7%). The median age of patients dying for SARS-CoV-2 infection was more than 30 years higher as compared with the national sample diagnosed with SARS-CoV-2 infection (median age 48 years). Women dying for SARS-CoV-2 infection had an older age than men (median age women, 86 years - median age men, 80 years) (https://www.epicentro.iss.it/en/coronavirus/bollettino/Report-COVID 2019_27_january_2021.pdf).

Under these premises, older persons are surely at increased risk for severe COVID-19, but age per se may not be the main contributor to the fatal outcome.

In this regards, the concept of frailty has been indicated as a way of capturing the biological aging of the individual. Frailty is one of the most debilitating conditions affecting older adults worldwide. It enhances the risk for both disability and premature mortality and is characterized by an increased vulnerability of physiological systems to external stressors [1]. Although human physiological systems have an inbuilt reserve to cope with stressors, aging reduces this physiological reserve [2]. The ability or not to respond to stress draws the different trajectories that characterize the aging of each person and that cause chronological age to differ from biological age.

COVID-19 is not exclusively gerophile (géro “old” and philia “love”) as young people can be infected, but generally, older people experience more severe symptoms and mortality. Thus, it is very likely that frailty, together with comorbidities, may contribute to the high vulnerability and increased risk of death of older people with SARS-CoV-2 infection.

Interestingly, in an Italian retrospective observational study, frailty has independently been associated with mortality in patients affected by COVID-19 and added prognostic information beyond chronological age in those aged 70 years or older [3].

In support, a recent meta-analysis described that the increase in clinical frailty score was positively associated with the increase of mortality outcome in patients with COVID-19 [4].

### Hints from centenarians

The experience of centenarians is crucial for understanding the mechanisms that regulate aging and age-related conditions. Centenarians are persons characterized by an exclusive signature that unequivocally identifies them, resulting from the interaction between genes and environmental factors, and from the combination of external stimuli met lifelong and individual genetic background. They are characterized by extremely heterogeneous health status and different degree of frailty, compared to a very narrow range of chronological age [5].

Albeit in centenarians the onset and the persistence of the age-related diseases undergo a compression towards the end of life [6], some of them had long histories of an age-related disease. Therefore, centenarians are persons probably with an extraordinary adaptive capacity and/or unusual functional reserve, that develop the ability to live with a debilitating (but not fatal) disease, delaying disability and death for even some decades [7–11].

The strength of centenarians derives from their experience encompassing more than a century, a period where enormous changes occurred worldwide, including two wars, lifestyle and nutrition, vaccination, antibiotics, kind of work, house heating. Thus, the centenarians’ phenotype is highly dynamic other than heterogeneous and more able to cope the stress.

In the era of COVID-19, it is interesting to underline that there are centenarians that showed a remarkable capacity to recover after coronavirus infection. In this regard, there are anecdotal observations that centenarians and sometime supercentenarians (people over 110 years old) survived and recovered after SARS-CoV-2 infection [12], as confirmed in a group of centenarians belonging to “Centenari a Trieste (CaT) study” [13].

### Biological mechanisms in centenarians

A crucial key factor contributing to the frailty development is the decline of the immune system during aging [14]. Some findings have identified inflammation as a feature playing a critical role in the pathophysiology of frailty in women more than in men [15, 16]. It is well known that the aging process is identified by a progressive decline in immunity and a deregulated inflammatory response [17, 18], partly justified by the existence of specific gene pathways associated with longevity that differ in males and females [19].

Inflammation is one of the pillar shared between aging and age-related diseases [20] and inflammaging identifies the low-grade inflammation that is typical of the older persons. It is caused by an increased stimulation of the innate immune response and by an accumulation of senescent cells that showed a pro-inflammatory secretory profile [21, 22].

These signatures may be expected to depend on the circumstances and immunological history of the individual. This immune decline can be a predisposing condition sustaining serious COVID-19 complications and explaining high mortality rates in older patients affected by COVID-19.

Common effects of aging on the adaptive immune system include a decline in the production of fresh naïve T cells, less expansive T cell receptor (TCR) repertoire, T cell metabolic dysfunction and weaker activation of T cells [23, 24].

Interestingly, one study found that supercentenarians tend to have an unusual population of cytotoxic CD4+ T cells (CTLs) that can take on the functions usually performed by CD8+ T cells and have a capability of activation that does not decline with age [25]. The CTLs seem to use the CD8 transcriptional program internally, while retaining CD4 expression on the cell surface. Indeed, upon ex vivo stimulation, CD4 CTLs from supercentenarians produced interferon γ (IFN-γ) and Tumour necrosis factor α (TNF-α) reinforcing their cytotoxic ability [25] and playing a key role in antiviral immunity.

Recent studies demonstrated that impaired type I IFN response may be a hallmark of severe COVID-19 [26–28].

Interestingly inactivating autoantibodies against type I IFN, that correlate with low serum of IFN-α concentrations, were found in up to 14% of patients with life-threatening COVID-19, while these autoantibodies were not present in patients with asymptomatic or mild COVID-19 [29].

The immune traits related to lifelong immune responsiveness and immunosenescence (e.g. level of circulating immunoglobulins and the peripheral CD4/CD8 T lymphocyte ratio) appear to be under strict genetic control [30]. Genes involved in immunity are highly polymorphic resulting from an evolutionary adaptation of the organism [31].

Among these genes, HLA are those more associated with human longevity due to their central role in the development of the adaptive immune response and of the individual’s response to life threatening diseases. The association of HLA genes with longevity has been described as population and gender specific [32].

Interestingly, the different genetic profiles of both HLA Class I and Class II play an important cell-mediated and humoral antiviral response, respectively. They potentially alter the transmission and the course of several infectious diseases.

Nowadays, contrasting results are reported regard the role of HLA in SARS-CoV-2 infection, probably due to the different experimental approaches adopted [33]. Moreover, the risk or the protection exerted by each allele of the highly polymorphic HLA genes could be masked by the other alleles [34].

However, it has been demonstrated that SARS-CoV-2 derived peptides processed in human cells, are presented by HLA class I molecules inducing SARS-CoV-2 specific cytotoxic innate immune response [35]. The different alleles of the HLA genes show different affinity to bind virus derived peptides, maybe causing a different risk to develop COVID-19 [35].

For example, the HLA haplotype “HLA-A*02.01 g-B*18.01 g-C*07.01 g-DRB1*11.04 g” has been suggested to be more protective towards SARS-CoV-2 infection in Italy [36].

Interestingly, the allele HLA-DRB1*11 was reported to be associated with longevity in Caucasian older women [37–39], whereas HLA-C*0701 has been described as a specific marker for longevity in men [31].

**Figure 1:**
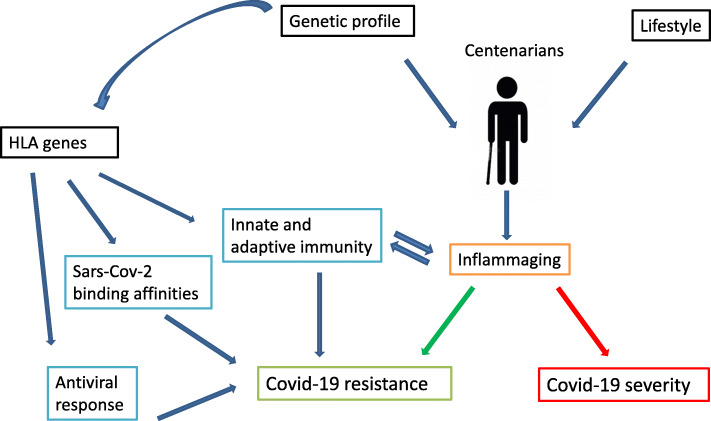
Centenarian’s portfolio and biological mechanisms underlying the coronavirus infection. Centenarians are persons characterized by an exclusive signature resulting from the interaction between genes and environmental factors. Inflammaging identifies the low-grade inflammation status typical of the older persons that seems to drive the course of the infection. HLA genes play a crucial role in immune response and antiviral reactivity and the HLA alleles show a specific binding affinity with Sars-Cov-2 peptides

Under these premises, the data from the HLA genetic profiles of centenarians may be useful to unravel the relationship between HLA alleles and COVID-19 development (Fig. 1).

## Implications of the hypothesis

It should be noted that we have to consider some social and behavioural aspects that may have enabled centenarians to cope and/or escape COVID-19.

It is known that there are multiple routes to achieve an exceptional longevity. In fact, centenarians can be categorized in survivors, delayers and escapers from the major diseases because of their heterogeneous phenotypes and probably genotypes [40]. It is also true that this notable heterogeneity comes from lifestyle habits and environmental factors, which may influence the centenarians’ capacity to survive to COVID-19.

Indeed, centenarians, due to their objective functional deficits, live more distancing from the other persons compared to seventy and/or eighty years old subjects, and at the same time may differently interact with environmental and lifestyle factors than normal people.

Overall, in this hypothesis, we assume that centenarian’s portfolio could help in identifying protective biological mechanisms that play a role in surviving or recovering from virus. In particular, the biology of centenarians could unravel doubts regarding the role of HLA mechanisms in COVID-19 infection.

In conclusion, we suggest to more deeply investigate the HLA genes as immunogenetic markers informative of the fatal outcome of the COVID-19 as well as of the different rate of severity.

With these premises, several studies evaluating the HLA binding affinity with the virus peptides together with assays to test T cell response were conducted as a promising strategy for the development of new vaccines [41].

A better understanding of patterns underlying risk of COVID-19 is mandatory in searching personalized treatments and new strategies.

## Data Availability

Not applicable.
